# Diagnostic Accuracy of Immunochromatographic Tests for the Detection of Norovirus in Stool Specimens: a Systematic Review and Meta-Analysis

**DOI:** 10.1128/spectrum.00467-21

**Published:** 2021-07-07

**Authors:** Seo Hee Yoon, Hye Rim Kim, Jong Gyun Ahn

**Affiliations:** a Department of Pediatrics, Severance Children’s Hospital, Yonsei University College of Medicine, Seoul, Republic of Korea; b Biostatistics Collaboration Unit, Department of Biomedical Systems Informatics, Yonsei University College of Medicine, Seoul, Republic of Korea; Broad Institute

**Keywords:** diagnosis, immunochromatographic tests, meta-analysis, noroviruses, rapid tests, systematic review

## Abstract

Noroviruses are the leading cause of acute gastroenteritis in all age groups and constitute a major health and economic burden worldwide. This systematic review and meta-analysis aimed to determine the diagnostic accuracy of immunochromatographic tests (ICTs) for the detection of norovirus in stool specimens, which has not been performed previously. In this systematic review and meta-analysis (registered on PROSPERO, CRD42020186911), we searched Medline/PubMed, Embase, Cochrane Library, and Web of Science for all studies published up to 16 May 2020. The values for sensitivity, specificity, positive likelihood ratio (LR+), negative likelihood ratio (LR−), and diagnostic odds ratio (DOR) of ICTs with 95% confidence interval (CI) were pooled using a bivariate random-effects model. The summary receiver operating characteristic curve and area under the curve were used to summarize overall test accuracy. We included 43 studies describing 7,428 samples. The overall estimates of sensitivity, specificity, LR+, LR−, DOR, and accuracy of ICT for diagnosing norovirus were 0.61 (95% CI, 0.54 to 0.67), 0.97 (95% CI, 0.95 to 0.98), 17.08 (95% CI, 11.15 to 26.18), 0.40 (95% CI, 0.34 to 0.46), 53.9 (95% CI, 31.32 to 92.78), and 0.928, respectively. Significant differences in pooled sensitivities were noted between age groups and in pooled DOR and LR+ between genogroups of included samples. ICT provides low sensitivity but high specificity and accuracy for detecting norovirus. Thus, an ICT for norovirus can be a rapid and convenient way for identifying patients early; however, a negative result cannot rule out norovirus infection and should be confirmed by a reference test.

## INTRODUCTION

Norovirus is the leading cause of acute gastroenteritis in all age groups (1, 2) and constitutes a major health and economic burden worldwide (3). Globally, it causes acute gastroenteritis in approximately 20% to 24% of community or outpatient clinic patients and 17% of hospitalized patients and 70,000 to 210,000 deaths annually (2, 4–7). Noroviruses are small, nonenveloped, positive-sense single-stranded RNA viruses that belong to the family *Caliciviridae* (8). Noroviruses can be classified into 10 genogroups (GI to GX) according to the amino acid sequence diversity of the major capsid protein VP1 and can further be divided into 49 genotypes (9). So far, GI, GII, GIV, GVIII, and GIX are known as human pathogens (9, 10). Noroviruses are extremely contagious because of their low infectious doses (≥18 viral particles) (11). Human noroviruses are transmitted primarily through the fecal-oral route, either through contaminated water or food, direct person-to-person contact, or fomites and airborne droplets from vomit (11, 12). These characteristics enable norovirus to spread rapidly (13). Additionally, some populations are at high risk of norovirus infection and severe complications: young children, the elderly, those dwelling in group settings (e.g., military), and immunocompromised individuals. Rapid detection of norovirus enables prevention and control of the outbreak, particularly for the high-risk group (14). Furthermore, prompt diagnosis of norovirus infection could be specifically helpful during the coronavirus disease pandemic because diarrhea is a common symptom of severe acute respiratory syndrome coronavirus-2 infection (15), which can also be an initial presenting symptom of norovirus infection.

Currently, the gold standard for norovirus diagnosis is reverse transcription-PCR (RT-PCR) or real-time RT-PCR (rRT-PCR) (8). However, being technically demanding, time consuming, and expensive, their use is limited to the patient ward or outpatient setting (16). The immunochromatographic test (ICT), also known as lateral flow assay, is one of the most popular point-of-care tests with antigen-antibody reactions on the membrane (17–21). ICT has several advantages such as simplicity, low cost, portability, and rapid assay time (within 5 to 15 min) and provides dichotomous answers detected using the naked eye (21–25). ICT targeting norovirus antigen has been developed and commercially used. However, systematic research on the performance of ICT for detecting norovirus in stool specimens has not yet been conducted. Thus, we performed this meta-analysis to synthesize and establish the diagnostic accuracy of ICT for norovirus infection.

## RESULTS

**FIG 1:**
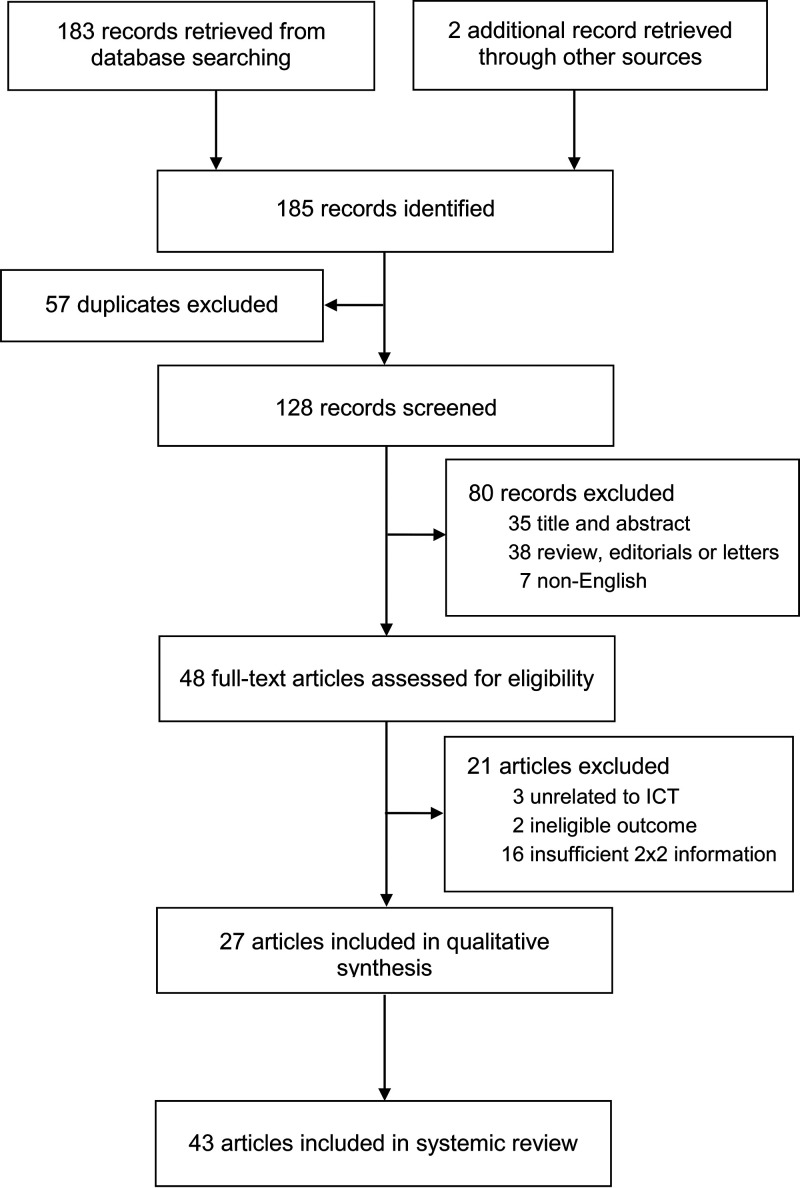
Flow diagram demonstrating study selection.

Overall, 185 articles were retrieved, and 48 articles underwent full-text review after removing duplicates and excluding articles based on the titles and abstracts. Twenty-one studies were excluded: 16 studies did not provide sufficient data for 2 by 2 contingency tables, two studies were not regarding norovirus, and the other three studies did not use ICT as the index test. The remaining 27 articles were eligible for data extraction, of which eight articles comprising 24 different sets of data using different brands of index tests or types of specimens (e.g., frozen or unfrozen) were provided with full 2 by 2 table information; thus, they were regarded as separate studies. Therefore, 43 studies comprising 7,428 samples were finally included in the systematic review and meta-analysis (Fig. 1) (16, 26–51).

The summary characteristics of all the included studies are demonstrated in Table 1. Studies were published between 2003 and 2020 and were conducted in the following countries: France (*n* = 7), Japan (*n* = 7), Thailand (*n* = 7), Australia (*n* = 5), and the United Kingdom (*n* = 5). Fourteen studies (32.6%) included only children, four studies (9.3%) included only adults, and three studies (7.0%) included both adults and children. The remaining studies (*n* = 22, 51.2%) did not specifically describe the age of the included participants. Most of the samples used in the studies included both GI and GII (*n* = 30, 69.8%), except one study (2.3%) that did not demonstrate the included genogroup; 11 studies included only GII (25.6%), and one study included only GI (2.3%). The number of studies in which frozen samples were used for both the index test and reference standard was 14 (32.6%), and the number with unfrozen samples was four (9.3%). The remaining studies (58.1%) used mixed samples or did not specify whether the samples were frozen or not. Among the ICTs, RIDA QUICK (R-Biopharm AG, Darmstadt, Germany) was assessed most frequently in the included studies (*n* = 19, 44.2%), consisting of 3,095 samples. Regarding the reference standard, most studies (*n* = 26, 60.5%) used RT-PCR as the reference standard, followed by rRT-PCR (*n* = 15, 34.9%) and RT-nested PCR (*n* = 2, 4.7%). Most studies did not describe the duration of symptoms before testing.

**TABLE 1:** Characteristics of the studies included in the meta-analysis

Study year and identifier (reference)	Country	Population	Included genogroup	Brand of index test	Specimen of ICT	Specimen of Reference test	Reference test	No. of:^*a*^			
								TP	FP	FN	TN
2003 Okame (26)	Japan	Children	NA^*b*^	IC assay	Stool (NA)	Stool (NA)	RT-PCR	24	2	9	20
2007 Nguyen (27)	Vietnam	Children	GII	NVIC-1 sticks, lot 0609	Unfrozen stool	Frozen stool	RT-PCR	14	0	5	85
2008 Khamrin (28)	Japan	Children	GI and GII	IC assay	Stool (NA)	Stool (NA)	RT-PCR	90	14	24	375
2008 Takanashi (29)	Japan	Children	GII	IC assay	Unfrozen stool	Unfrozen stool	RT-PCR	30	4	13	60
2009 Khamrin (30)	Japan	Children	GII	IC assay	Stool (NA)	Stool (NA)	RT-PCR	46	0	15	14
2009 Mutoh-1 (31)	Japan	Adults	GI and GII	Quick Ex-Norovirus	Mixed stool	Mixed stool	RT-PCR	9	1	9	15
2009 Mutoh-2 (31)	Japan	Children	GII	Quick Ex-Norovirus	Mixed stool	Mixed stool	RT-PCR	15	2	11	25
2010 Bruins (32)	Netherlands	NA	GI and GII	RIDA QUICK	Stool (NA)	Stool (NA)	Real-time RT-PCR	56	4	42	435
2010 Kirby (33)	Brazil	Children	GI and GII	RIDA QUICK	Stool (NA)	Stool (NA)	RT-PCR	66	1	30	59
2010 Thongprachum (34)	Thailand	Children	GII	IP-NoV	Stool (NA)	Stool (NA)	RT-PCR	46	2	16	399
2011 Bruggink (35)	Australia	NA	GI and GII	RIDA QUICK	Frozen stool	Frozen stool	RT-PCR	83	0	17	93
2012 Battaglioli-1 (36)	USA	NA	GI	RIDA QUICK	Mixed stool	Mixed stool	Real-time RT-PCR	8	0	11	18
2012 Battaglioli-2 (36)	USA	NA	GII	RIDA QUICK	Mixed stool	Mixed stool	Real-time RT-PCR	19	0	6	18
2012 Kim (37)	Republic of Korea	Children and adults	GI and GII	SD BIOLINE Norovirus	Frozen stool	Frozen stool	Real-time RT-PCR	83	0	9	126
2012 Park (38)	Republic of Korea	Children and adults	GII	SD BIOLINE Norovirus	Stool (NA)	Frozen stool	Real-time RT-PCR	52	1	16	342
2012 Pombubpa-1 (39)	Thailand	NA	GI and GII	RIDA QUICK	Stool (NA)	Frozen stool	RT-PCR	15	4	3	28
2012 Pombubpa-2 (39)	Thailand	NA	GI and GII	RIDA QUICK	Stool (NA)	Frozen stool	RT-nested PCR	26	4	28	28
2012 Pombubpa-3 (39)	Thailand	NA	GI and GII	RIDA QUICK	Stool (NA)	Frozen stool	Real-time RT-PCR	23	3	8	20
2013 Ambert-Balay-1 (40)	France	NA	GI and GII	RIDA QUICK	Frozen stool	Frozen stool	RT-PCR	113	0	105	62
2013 Ambert-Balay-2 (40)	France	NA	GI and GII	ImmunoCard STAT!	Frozen stool	Frozen stool	RT-PCR	62	0	113	26
2013 Ambert-Balay-3 (40)	France	NA	GI and GII	NOROTOP+	Frozen stool	Frozen stool	RT-PCR	76	0	72	25
2013 Ambert-Balay-4 (40)	France	NA	GI and GII	SD BIOLINE Norovirus	Frozen stool	Frozen stool	RT-PCR	78	0	111	22
2013 Ambert-Balay-5 (40)	France	NA	GI and GII	RIDA QUICK	Unfrozen stool	Unfrozen stool	RT-PCR	29	0	12	33
2013 Bruggink-1 (41)	Australia	NA	GI and GII	SD BIOLINE Norovirus	Unfrozen stool	Unfrozen stool	RT-PCR	23	3	27	213
2013 Bruggink-2 (41)	Australia	NA	GI and GII	SD BIOLINE Norovirus	Frozen stool	Frozen stool	RT-PCR	62	0	38	99
2013 Bruggink-3 (41)	Australia	NA	GI and GII	SD BIOLINE Norovirus	Unfrozen stool^*c*^	Unfrozen stool^*c*^	RT-PCR	54	0	46	99
2013 Kas (42)	Papua New Guinea	Children	GI and GII	IP-Triple I	Frozen stool	Frozen stool	Real-time RT-PCR	2	1	17	179
2015 Bruggink (43)	Australia	NA	GI and GII	RIDA QUICK (N1402)	Frozen stool	Frozen stool	RT-PCR	87	3	13	96
2015 Vyas-1 (44)	UK	NA	GI and GII	RIDA QUICK	Frozen stool	Unfrozen stool	Real-time RT-PCR	57	0	40	4
2015 Vyas-2 (44)	UK	NA	GI and GII	Immunoquick	Frozen stool	Unfrozen stool	Real-time RT-PCR	29	1	68	3
2015 Vyas-3 (44)	UK	NA	GI and GII	Noroscreen	Frozen stool	Unfrozen stool	Real-time RT-PCR	22	0	75	4
2015 Vyas-4 (44)	UK	NA	GI and GII	NOROTOP+	Frozen stool	Unfrozen stool	Real-time RT-PCR	29	0	68	4
2015 Vyas-5 (44)	UK	NA	GI and GII	ImmunoCard STAT!	Frozen stool	Unfrozen stool	Real-time RT-PCR	23	1	74	3
2016 Hosoda (45)	Japan	Adults	GII	ImmunoCatch-Noro	Unfrozen stool^*d*^	Frozen stool^*d*^	RT-PCR	19	6	16	53
2016 Sharaf (46)	Egypt	Children	GI and GII	RIDA QUICK	Frozen stool	Frozen stool	RT-nested PCR	46	3	8	143
2017 Gaspard-1 (47)	France	Adults	GI and GII	RIDA QUICK (N1403+N1402)	Unfrozen stool	Frozen stool	RT-PCR	27	1	22	20
2017 Gaspard-2 (47)	France	Adults	GII	RIDA QUICK (N1403+N1402)	Unfrozen stool	Frozen stool	RT-PCR	1	0	4	39
2017 Jonckheere (16)	Belgium	Children and adults	GI and GII	RIDA QUICK (N1402)	Unfrozen stool	Frozen stool	Real-time RT-PCR	91	3	34	643
2017 Kumthip-1 (48)	Thailand	Children	GI and GII	RIDA QUICK (N1402)	Frozen stool	Frozen stool	RT-PCR	32	0	0	8
2017 Kumthip-2 (48)	Thailand	Children	GI and GII	RIDA QUICK (N1402)	Frozen stool	Frozen stool	Real-time RT-PCR	32	0	0	8
2018 Khamrin (49)	Thailand	Children	GII	IP Line Duo Noro-Rota	Stool (NA)	Stool (NA)	RT-PCR	34	0	4	17
2018 Sakalkina (50)	Russia	NA	GI and GII	RIDA QUICK	Frozen stool	Frozen stool	Real-time RT-PCR	11	0	79	30
2020 Shaha (51)	Bangladesh	Children	GII	IP Line Duo Noro-Rota	Frozen stool	Frozen stool	RT-PCR	10	1	0	89

a TP, true positives; FP, false positives; FN, false negatives; TN, true negatives.

b NA, not available.

c Samples were tested more than three days after collection and within 30 days.

d Samples were taken in a cup (bulk stools) or via rectal swab.

**FIG 2:**
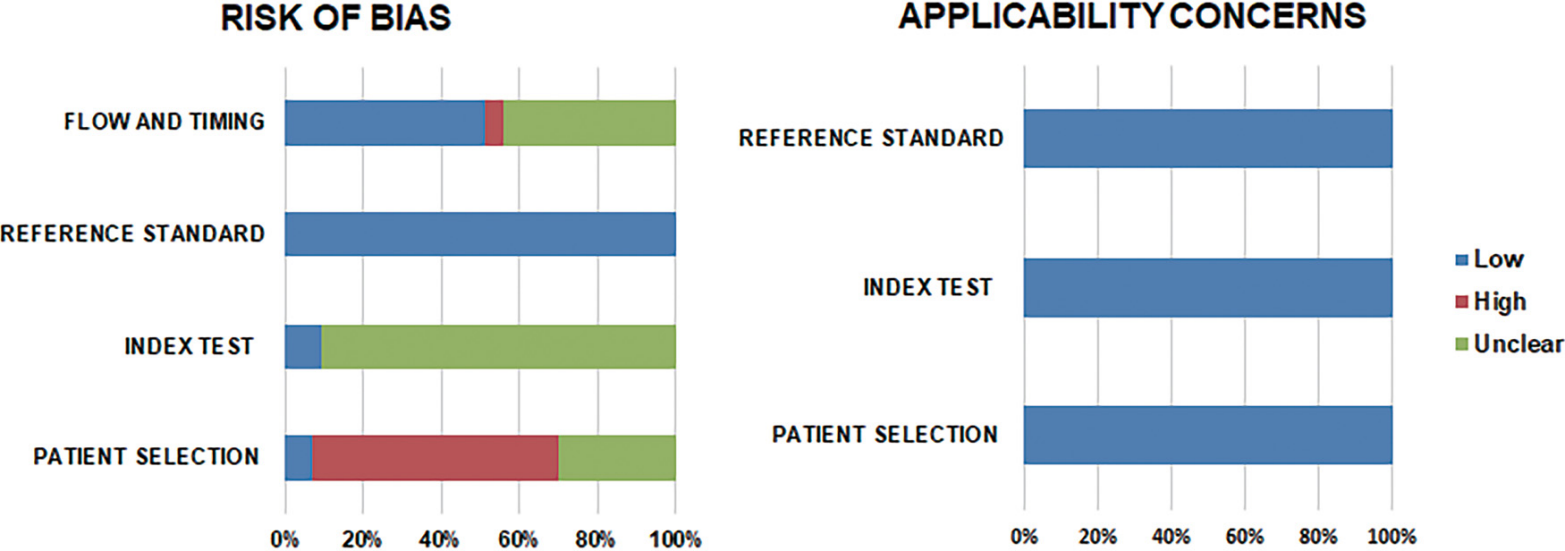
Quality assessment of enrolled studies using the Quality Assessment of Diagnostic Accuracy Studies 2 (QUADAS-2) tool.

The methodological quality of the included studies is demonstrated in Fig. 2. Regarding the risk of bias in patient selection, 62.8% of the studies had a “high” risk of bias because most studies did not clarify how they enrolled patients, whether consecutively or randomly; additionally, a diagnostic case-control design (e.g., norovirus infection status was already revealed using the reference standard, and samples were subsequently selected before the index test was performed) was used (52). Most studies (90.7%) had an “unclear” risk of bias in the index test domain, since the blinding of personnel who conducted the index test to the results of the reference test was not clarified. All studies were scored as “low” risk of bias, since RT-PCR or rRT-PCR was used for diagnosing norovirus infection. Regarding the flow and timing domain, we scored as “low” risk of bias if the interval between the index test and reference test was within 24 h. Half of the studies (51.2%) were at “low” risk and 44.2% were at “unclear” risk, since the authors did not clarify the specific interval time. Applicability of studies was scored as low concern for all studies.

**FIG 3:**
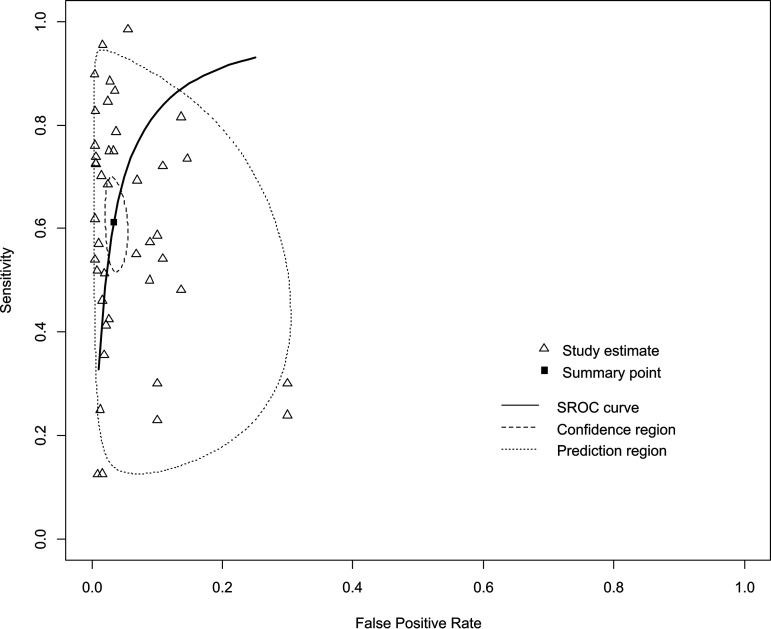
Summary receiver operating characteristic (SROC) curves of the diagnostic accuracy of immunochromatographic tests (ICT) for norovirus infection. Summary points of the sensitivity and specificity, SROC curve, 95% confidence region, and 95% prediction region are provided. The area under the curve of the SROC for ICT was 0.928.

The sensitivities and specificities of the included studies ranged from 0.105 to 1.00 and from 0.750 to 1.00, respectively (see Fig. S1 and S2 and Table S1 in the supplemental material). The overall sensitivity of each study estimated from the bivariate random-effects model was 0.609 (95% confidence interval [CI], 0.542 to 0.673), and the specificity was 0.967 (95% CI, 0.951 to 0.978). The diagnostic odds ratio (DOR) was 53.901 (95% CI, 31.316 to 92.776). The positive likelihood ratio (LR+) and negative likelihood ratio (LR−) were 17.082 (95% CI, 11.145 to 26.182) and 0.399 (95% CI, 0.343 to 0.464), respectively (Table 2; see also Table S1). The area under the curve (AUC) of the summary receiver operating characteristic (SROC) curve was 0.928 (Fig. 3). The symmetrical funnel plot, Begg’s test (*P* = 0.645), and Egger’s regression test (*P* = 0.281) revealed no publication bias (see Fig. S3).

**TABLE 2:** Subgroup analysis of the studies

Group	*n*	Value (95% CI) or *P* value				
		Sensitivity	Specificity	DOR	LR+	LR−
Overall	43	0.609 (0.542–0.673)	0.967 (0.951–0.978)	53.901 (31.316–92.776)	17.082 (11.145–26.182)	0.399 (0.343–0.464)
Population						
NA^*a*^	22	0.511 (0.429–0.593)	0.960 (0.926–0.978)	29.520 (13.391–65.075)	11.754 (5.979–23.106)	0.486 (0.418–0.566)
Adults	4	0.525 (0.432–0.617)	0.912 (0.845–0.952)	11.595 (5.270–25.513)	5.860 (3.127–10.979)	0.569 (0.448–0.721)
Children	14	0.744 (0.660–0.813)	0.969 (0.950–0.981)	96.275 (46.905–197.607)	21.648 (13.231–35.419)	0.253 (0.163–0.392)
Children and adults	3	0.801 (0.675–0.886)	0.995 (0.989–0.998)	630.095 (259.035–1532.688)	151.943 (65.781–350.965)	0.202 (0.122–0.333)
* P* value		0.0136	0.2741	0.427	0.3196	0.0675
Included norovirus genogroup						
NA	1	0.721 (0.549–0.845)	0.891 (0.688–0.968)	21.147 (4.664–95.888)	6.582 (2.009–21.562)	0.311 (0.179–0.541)
GI	1	0.425 (0.233–0.642)	0.974 (0.690–0.998)	27.348 (1.438–520.211)	16.128 (1.000–260.056)	0.590 (0.407–0.854)
GII	11	0.711 (0.635–0.777)	0.975 (0.942–0.989)	98.898 (31.079–314.705)	27.201 (11.026–67.102)	0.322 (0.236–0.441)
GI and GII	30	0.575 (0.494–0.653)	0.966 (0.946–0.979)	46.232 (23.825–89.712)	14.954 (8.772–25.492)	0.428 (0.366–0.500)
* P* value		0.7679	0.0526	0.019	0.0362	0.6749
Type of specimen						
NA	25	0.595 (0.507–0.676)	0.953 (0.922–0.973)	31.976 (15.271–66.956)	11.756 (6.447–21.437)	0.4157 (0.344–0.502)
Frozen	14	0.650 (0.510–0.768)	0.981 (0.969–0.989)	152.193 (66.842–346.532)	33.128 (19.888–55.182)	0.3670 (0.283–0.476)
Unfrozen	4	0.593 (0.480–0.698)	0.976 (0.927–0.992)	49.688 (23.110–106.834)	21.469 (8.737–52.755)	0.4263 (0.333–0.546)
* P* value		0.1537	0.9736	0.3525	0.6261	0.1385
Brand						
RIDA QUICK	19	0.662 (0.565–0.746)	0.967 (0.940–0.982)	72.1157 (33.514–155.180)	19.2199 (10.2994–35.8666)	0.3461 (0.2634–0.4549)
Other	24	0.568 (0.477–0.654)	0.967 (0.945–0.980)	43.0892 (19.971–92.968)	15.4620 (8.368–28.570)	0.4404 (0.3737–0.5190)
* P* value		0.1537	0.9736	0.3525	0.6261	0.1385
Reference test						
Real-time RT-PCR	15	0.513 (0.368–0.655)	0.968 (0.922–0.987)	33.172 (9.290–118.445)	12.492 (4.241–36.800)	0.483 (0.386–0.606)
RT-PCR	28	0.657 (0.590–0.718)	0.963 (0.946–0.975)	63.773 (36.525–111.349)	17.923 (11.80–27.222)	0.370 (0.318–0.430)
* P* value		0.0745	0.8078	0.3565	0.5414	0.0537

a NA, not available.

Substantial heterogeneity was found in both sensitivity (*I*^2^ = 90.9% [95% CI, 88.6% to 92.7%]; *P* value of Q, <0.001) and specificity (*I*^2^ = 61.9% [95% CI, 47.0% to 72.6%]; *P* value of Q, <0.001) (Fig. S1 and S2). We investigated the potential sources of heterogeneity using subgroup analysis and meta-regression for DOR. The summary estimates for each subgroup are presented in Table 2. Significant differences in pooled sensitivities were noted between the population’s ages; pooled DORs and LR+ values between genogroups of included samples. The frozen/thawing step and the brand of index test (e.g., RIDA QUICK versus others) did not affect the performance of the ICT (Table 2). In meta-regression analysis, the population’s age and type of specimen were a significant source of heterogeneity (*P* < 0.05) (Table S2).

## DISCUSSION

To our knowledge, this is the first systematic review with meta-analysis evaluating the diagnostic performance of ICT for norovirus infection. Our review demonstrates that ICT possesses low sensitivity (0.609) but high specificity (0.967) and diagnostic accuracy (0.928) for the detection of norovirus in stool specimens. These results indicate that a positive test result is unlikely to be false positive (40, 53). Therefore, with a positive ICT result in a patient with symptoms, physicians can diagnose norovirus infection with conviction and can initiate appropriate infection control management. However, a negative ICT result cannot rule out the presence of norovirus definitively (53) and needs to be further confirmed using a reference standard test if test results can affect the decision of individual management. The following are the principal advantages of an ICT assay: inexpensive, rapid testing, and accessibility without the need for special skills and equipment (48). These advantages enable early and easy diagnosis in emergency rooms or resource-limited settings, such as private clinics and nursing homes (8, 54, 55). Furthermore, ICT can be useful in situations where multiple samples need to be tested, such as an outbreak setting (56).

Considerable heterogeneity in the performance of ICTs existed in our analysis. Among the presumed factors, age groups and norovirus genogroups showed significant differences in the performance of ICTs. However, the numbers of studies within each subgroup were low, and several samples were categorized in the not available (NA) group owing to limited information; thus, the results should be carefully interpreted.

The population’s age was also one of the significant heterogeneity sources in meta-regression analysis. In a previous hospital-based, 2-year observational study, Chan et al. (57) reported that norovirus load inversed with increasing age: young children (≤5 years) showed significantly higher viral loads for both GII.4 and non-GII.4 than older children (>5 years). The authors suspected that a previous norovirus infection might have resulted in partial immunity (57). This difference in viral load concentration might have led to the difference in sensitivity between different age groups. Additionally, clinical studies evaluating ICT focusing on the adult population (*n* = 4) were also limited in our analysis; thus, further studies evaluating the accuracy of ICT between different age groups are required. Moreover, most studies did not demonstrate the influence of age among the included patients (*n* = 22, 51.2%), which limits the ability to fully explain study heterogeneity based on age groups.

Interestingly, an increase in the sensitivity of ICT through the freeze-thaw process has been reported by several studies (29, 35, 40, 41). It is speculated the freeze-thaw step might expose the inner epitopes and eliminate inhibitors from stool suspension (29). The sample type, whether frozen or unfrozen, was another significant heterogeneity factor in the meta-regression; however, pooled sensitivity and specificity of ICTs were not significantly different in the subgroup analysis. Again, there was a limitation that most studies (*n* = 25, 58.1%) used a mixture of frozen and nonfrozen samples or the unification of the index test and reference test samples were not achieved; thus, the results should be interpreted carefully.

There were significant differences in pooled LR+ and DOR of ICT values between genogroups. Especially, pooled LR+ (27.20) and DOR (98.89) of ICT for GII showed the highest value. In several previous studies, the RIDA QUICK ICT test did not detect GI; however, only a few specimens were included (GI, *n* <10) (33, 35). Battaglioli et al. (36) reported that the sensitivity of ICT for GI (*n* = 37) was 42.1%, and the sensitivity for GII (*n* = 43) was 76%, whereas the specificity for both was 100%. Although the sensitivity of ICT for GI was low in previous studies, the sensitivity for GII was generally fair; GII (mostly the GII.4 genotype) has been the most common genogroup worldwide followed by GI and GIV (58–62). Specifically, the prevalence of GII accounts for approximately 96%, GI accounts for 3.6%, and mixed infections of both GI and GII account for 0.4% in children (63). A recent review of studies published from 1997 to 2018 that detected norovirus genogroups in eight low-income and 21 low-to-middle-income countries revealed that the prevalence of norovirus GII was much higher than that of GI in symptomatic infections (83% to 87% versus 12% to 13%) (64); thus, ICT can still be considered useful in clinical practice (36).

Most index tests included in our review used a commercially available kit, the RIDA QUICK (44.2%), and the performance of ICTs was not significantly different between other ICT brands. There were two head-to-head studies comparing commercial ICT kits (40, 44). Ambert-Balay et al. (40) evaluated four ICT kits: RIDA QUICK, ImmunoCard STAT! (Meridian Bioscience Europe, Nice, France), NOROTOP+ (Pro-Lab Diagnostics, Bromborough, UK), and SD BIOLINE (Standard Diagnostics, Inc., Kyonggi-do, Republic of Korea). Among these four tests, RIDA QUICK showed the lowest sensitivity for the detection of norovirus GI on thawed samples (0.17) but showed the highest sensitivity (0.64) for GII. Furthermore, the sensitivity of RIDA QUICK was increased to 78% for the GII.4 strain, which is the most prevalent genotype globally. The specificities of all tests were 100%, with no cross-reactivity against other enteric viruses. Vyas et al. (44) also compared five commercial ICTs: RIDA QUICK, ImmunoCard STAT!, NOROTOP+, Immunoquick (Quadratech Diagnostics Ltd., Epsom, UK), and Noroscreen (Microgen Bioproducts Ltd., Camberley, UK). RIDA QUICK showed the highest overall sensitivity (0.59) and specificity (1.0). While the specificities were generally high, the RIDA QUICK showed poorer sensitivity for detecting GI than for detecting GII (33, 35, 36). The updated RIDA QUICK (N1402) assay is now available, which can detect broader genotypes successfully (e.g., GI.2, GI.4, GII.6, and GII.7) (48). The N1402 version has demonstrated high sensitivity of 0.73 to 1.0 and specificity of 0.97 to 1.0 (16, 43, 48) in several studies. Notably, the N1402 version detected 93% of GI and 98% of GII norovirus (43), which greatly increased the sensitivity for the GI strain.

In our review, the performances of ICTs, whether using RT-PCR or rRT-PCR as a reference standard, were not significantly different. Generally, rRT-PCR can detect and quantify norovirus genomes, which provides rapid results and reduces the risk of carryover contamination (38, 39). However, the sensitivity and specificity of rRT-PCR can vary, since noroviruses have very highly diverse genomes and different rRT-PCR protocols utilize different primers or probes and reagents and have different reaction conditions (65–68).

Our study has several limitations. First, most studies did not provide clinical data such as the comorbidity of included patients or the time interval between the onset of symptoms and testing. Second, we could not compare the pooled estimates of ICT performance between specific genotypes due to the limited number of clinical studies that provided those data. Third, we could not evaluate the effects of industrial sponsorship and blinding of testing through meta-analysis, because most included studies did not provide that information. Although several studies showed that the updated RIDA QUICK (N1402) assay can detect broader genotypes and increase the diagnostic performance for the GI strain, the subgroup analysis according to the RIDA QUICK versions was limited due to the insufficient number of studies and the studies that used different RIDA QUICK versions as an index test concomitantly. Further studies are required to ensure the applicability of the results of studies of ICTs conducted in various clinical settings and explore the performance of the N1402 version of the RIDA QUICK assay.

In conclusion, ICT is a simple, fast, and reliable method with low sensitivity, high specificity, and accuracy for norovirus detection. Thus, if clinicians are aware of false negativity, ICT could function as a good axillary modality to prevent norovirus outbreaks and could help make decisions for appropriate patient management.

## MATERIALS AND METHODS

### Search strategy and selection criteria.

This systematic review and meta-analysis was conducted according to the Preferred Reporting Items for Systematic Reviews and Meta-Analyses (PRISMA) statement (69) guidelines and was registered at the International Prospective Register of Systematic Reviews (PROSPERO; record CRD42020186911). A systematic search of PubMed, Embase, Cochrane Library database, and Web of Science was conducted with the keywords “norovirus,” “immunochromatography,” and “lateral flow assay” on 16 May 2020. Additional eligible studies were identified from the reference lists of the included studies. There were no date restrictions for the searches. Studies were included if they evaluated the accuracy of an ICT for the detection of norovirus and provided enough information for constructing a 2 by 2 table. Studies in which RT-PCR or rRT-PCR served as the reference standard were considered eligible. We included studies using rectal swabs when rectal swab specimens were properly collected in a standardized manner with a flocked swab and used as standard tool of a norovirus ICTs and other swab specimens were concomitantly collected and washed (soaked) in sterile saline or a transportation medium and then preserved properly for the reference standard, RT-PCR or rRT-PCR. Reviews, case reports, editorials, conference abstracts, letters, and *in vitro* or animal experiments were excluded.

Two independent reviewers (S.H.Y. and J.G.A.) undertook title and abstract screening, followed by full-text review. Disagreements between reviewers were resolved through discussion or arbitration by a third reviewer (H.R.K.). The author names, country, year of publication, included norovirus genogroup, population (children [aged ≤18 years] and adult [aged ≥19 years]), sample size, index test assay, reference standard, type of specimens (frozen or unfrozen or mixed [frozen with unfrozen] specimen for index and reference tests), and the values for true positive, false positive, true negative, and false negative were extracted. Samples that initially came out as false positives but were later found to be true positives using other RT-PCR protocols were counted as true positives. When articles included multiple study groups, each group was regarded as a separate study.

### Data analysis.

Pooled estimates of sensitivity, specificity, positive likelihood ratio (LR+), negative likelihood ratio (LR−), and diagnostic odds ratio (DOR) along with their 95% confidence intervals (CIs) were calculated using the bivariate random-effects models (70, 71). LR+ is the probability of positivity in a patient over the probability of positivity in a participant without the disease (72, 73). DOR is the ratio of the odds of positivity in participants with the disease to the odds of positivity in participants without the disease, with higher values indicating better discriminatory power of the diagnostic test (73, 74). The area under the curve (AUC) based on the summary receiver operating characteristic (SROC) curve was obtained to summarize the overall test accuracy. We added a fixed value (0.5) to all zero cells in 2 by 2 tables as a continuity correction.

Heterogeneity was evaluated using Cochran’s Q test (*P* < 0.1, significant heterogeneity), *I*^2^ metric (0% to 24%, low; 25% to 74%, moderate; and 75% to 100%, considerable heterogeneity), and visual inspection of forest plots. With significant heterogeneity, we conducted subgroup analysis and meta-regression analysis for assessing heterogeneity with 95% CIs using the following as covariates: age of the population (adults, children, and adults and children), genogroup (GI and GII versus GII), type of specimen (frozen versus unfrozen: we classified frozen specimen when both the index test sample and reference test sample were frozen; unfrozen specimen was defined when both samples were unfrozen), index test brand (RIDA QUICK versus others), and reference test (RT-PCR versus rRT-PCR) used. We classified RT-nested PCR as RT-PCR when performing meta-regression. Publication bias was assessed using the Begg’s test (75), Egger’s regression test (76), and asymmetry of funnel plots (77, 78). The Quality Assessment of Diagnostic Accuracy Studies 2 (QUADAS-2) tool was used for evaluating the validity of the included studies (79). The QUADAS-2 tool comprises four domains: patient selection, index test, reference standard, and flow and timing. The domains were assessed for risk of bias and applicability. Two authors independently performed the quality assessment, and disagreements between them were resolved by a consensus. R statistical software (version 3.4.3; R Foundation for Statistical Computing, Vienna, Austria) was used for performing meta-analyses. *P* values of <0.05 were considered statistically significant.
