# Supplementary material for: Diagnostic Accuracy of Immunochromatographic Tests for the Detection of Norovirus in Stool Specimens: a Systematic Review and Meta-Analysis
Source: Microbiol Spectr. 2021 Jul 7;9(1):10.1128/spectrum.00467-21. doi: 10.1128/spectrum.00467-21 (PMC8552764; doi:10.1128/spectrum.00467-21)
Supplement: Supplemental file 1 — Supplemental material. Download SPECTRUM00467-21_Supp_1_seq7.pdf, PDF file, 0.4 MB [file spectrum00467-21_supp_1_seq7.pdf]

TABLE S1. Summary estimates of the diagnostic accuracy

Study	Sensitivity [95% CI]	Specificity [95% CI]	DOR [95% CI]	LR+ [95% CI]	LR- [95% CI]
2003 Okame (1)	0.727 [0.545; 0.867]	0.909 [0.708; 0.989]	21.147 [4.664; 95.888]	6.629 [2.019; 21.766]	0.313 [0.179; 0.548]
2007 Nguyen (2)	0.737 [0.488; 0.909]	1.000 [0.958; 1.000]	450.818 [23.641; 8596.858]	124.7 [7.76; 2003.796]	0.277 [0.136; 0.564]
2008 Khamrin (3)	0.789 [0.703; 0.860]	0.964 [0.940; 0.980]	95.659 [48.039; 190.481]	21.166 [12.661; 35.387]	0.221 [0.156; 0.315]
2008 Takanashi (4)	0.698 [0.539; 0.828]	0.938 [0.848; 0.983]	30.374 [9.596; 96.144]	10.013 [4.019; 24.944]	0.33 [0.21; 0.516]
2009 Khamrin (5)	0.754 [0.627; 0.855]	1.000 [0.768; 1.000]	87 [4.897; 1545.512]	22.5 [1.469; 344.647]	0.259 [0.166; 0.402]
2009 Mutoh-1 (6)	0.500 [0.260; 0.740]	0.938 [0.698; 0.998]	10.333 [1.542; 69.225]	5.667 [1.152; 27.867]	0.548 [0.342; 0.88]
2009 Mutoh-2 (6)	0.577 [0.369; 0.766]	0.926 [0.757; 0.991]	13.748 [3.048; 62.006]	6.43 [1.885; 21.926]	0.468 [0.297; 0.736]
2010 Bruins (7)	0.571 [0.467; 0.671]	0.991 [0.977; 0.998]	128.658 [46.842; 353.374]	55.802 [21.909; 142.132]	0.434 [0.346; 0.544]
2010 Kirby (8)	0.688 [0.585; 0.778]	0.983 [0.911; 1.000]	86.486 [16.182; 462.233]	27.88 [5.707; 136.202]	0.322 [0.24; 0.434]
2010 Thongprachum (9)	0.742 [0.615; 0.845]	0.995 [0.982; 0.999]	450.345 [115.077; 1762.396]	118.686 [34.193; 411.962]	0.264 [0.174; 0.399]
2011 Bruggink (10)	0.830 [0.742; 0.898]	1.000 [0.961; 1.000]	892.257 [52.835; 15067.996]	155.426 [9.78; 2470.153]	0.174 [0.114; 0.267]
2012 Battaglioli-1 (11)	0.421 [0.203; 0.665]	1.000 [0.815; 1.000]	27.348 [1.438; 520.211]	16.15 [1; 260.894]	0.591 [0.402; 0.867]
2012 Battaglioli-2 (11)	0.760 [0.549; 0.906]	1.000 [0.815; 1.000]	111 [5.833; 2112.166]	28.5 [1.833; 443.184]	0.257 [0.131; 0.502]
2012 Kim (12)	0.902 [0.822; 0.954]	1.000 [0.971; 1.000]	2223.737 [127.706; 38721.694]	228.054 [14.33; 3629.233]	0.103 [0.056; 0.187]
2012 Park (13)	0.765 [0.646; 0.859]	0.997 [0.984; 1.000]	726.515 [133.185; 3963.084]	174.493 [35.149; 866.241]	0.24 [0.158; 0.366]
2012 Pombubpa-1 (14)	0.833 [0.586; 0.964]	0.875 [0.710; 0.965]	28.048 [6.087; 129.23]	5.982 [2.469; 14.493]	0.213 [0.082; 0.555]
2012 Pombubpa-2 (14)	0.481 [0.343; 0.622]	0.875 [0.710; 0.965]	5.889 [1.91; 18.16]	3.533 [1.435; 8.702]	0.6 [0.45; 0.801]
2012 Pombubpa-3 (14)	0.742 [0.554; 0.881]	0.870 [0.664; 0.972]	16.193 [4.08; 64.272]	5.036 [1.87; 13.558]	0.311 [0.171; 0.566]
2013 Ambert-Balay-1 (15)	0.518 [0.450; 0.586]	1.000 [0.942; 1.000]	134.479 [8.215; 2201.453]	65.301 [4.118; 1035.615]	0.486 [0.423; 0.558]
2013 Ambert-Balay-2 (15)	0.354 [0.284; 0.430]	1.000 [0.868; 1.000]	29.185 [1.749; 487.101]	19.176 [1.222; 300.936]	0.657 [0.582; 0.742]
2013 Ambert-Balay-3 (15)	0.514 [0.430; 0.596]	1.000 [0.863; 1.000]	53.814 [3.217; 900.325]	26.698 [1.708; 417.407]	0.496 [0.417; 0.59]
2013 Ambert-Balay-4 (15)	0.413 [0.342; 0.486]	1.000 [0.846; 1.000]	31.682 [1.894; 530.071]	19.005 [1.219; 296.327]	0.6 [0.525; 0.686]
2013 Ambert-Balay-5 (15)	0.707 [0.545; 0.839]	1.000 [0.894; 1.000]	158.12 [8.968; 2787.902]	47.762 [3.028; 753.413]	0.302 [0.189; 0.482]

Study	Sensitivity [95% CI]	Specificity [95% CI]	DOR [95% CI]	LR+ [95% CI]	LR- [95% CI]
2013 Bruggink-1 (16)	0.460 [0.318; 0.607]	0.986 [0.960; 0.997]	52.127 [15.841; 171.534]	28.569 [9.695; 84.188]	0.548 [0.425; 0.707]
2013 Bruggink-2 (16)	0.620 [0.517; 0.715]	1.000 [0.963; 1.000]	323.052 [19.496; 5353.146]	123.762 [7.762; 1973.308]	0.383 [0.299; 0.491]
2013 Bruggink-3 (16)	0.540 [0.437; 0.640]	1.000 [0.963; 1.000]	233.237 [14.096; 3859.253]	107.921 [6.758; 1723.529]	0.463 [0.374; 0.572]
2013 Kas (17)	0.105 [0.013; 0.331]	0.994 [0.969; 1.000]	17.095 [2.13; 137.236]	15.083 [2.102; 108.25]	0.882 [0.747; 1.042]
2015 Bruggink (18)	0.870 [0.788; 0.929]	0.970 [0.914; 0.994]	178.704 [53.251; 599.707]	24.752 [8.819; 69.472]	0.139 [0.084; 0.228]
2015 Vyas-1 (19)	0.588 [0.483; 0.687]	1.000 [0.398; 1.000]	12.778 [0.669; 243.955]	5.867 [0.421; 81.795]	0.459 [0.315; 0.668]
2015 Vyas-2 (19)	0.299 [0.210; 0.400]	0.750 [0.194; 0.994]	1.005 [0.141; 7.14]	1.003 [0.254; 3.959]	0.999 [0.554; 1.798]
2015 Vyas-3 (19)	0.227 [0.148; 0.323]	1.000 [0.398; 1.000]	2.682 [0.139; 51.73]	2.296 [0.161; 32.642]	0.856 [0.627; 1.169]
2015 Vyas-4 (19)	0.299 [0.210; 0.400]	1.000 [0.398; 1.000]	3.876 [0.202; 74.309]	3.01 [0.213; 42.471]	0.777 [0.564; 1.069]
2015 Vyas-5 (19)	0.237 [0.157; 0.334]	0.750 [0.194; 0.994]	0.736 [0.103; 5.268]	0.799 [0.2; 3.192]	1.086 [0.605; 1.948]
2016 Hosoda (20)	0.543 [0.366; 0.712]	0.898 [0.792; 0.962]	9.727 [3.42; 27.666]	5 [2.279; 10.969]	0.514 [0.357; 0.741]
2016 Sharaf (21)	0.852 [0.729; 0.934]	0.979 [0.941; 0.996]	224.294 [61.867; 813.164]	35.509 [12.535; 100.588]	0.158 [0.085; 0.294]
2017 Gaspard-1 (22)	0.551 [0.402; 0.693]	0.952 [0.762; 0.999]	16.704 [2.906; 96.021]	8.067 [1.687; 38.58]	0.483 [0.348; 0.669]
2017 Gaspard-2 (22)	0.200 [0.005; 0.716]	1.000 [0.910; 1.000]	26.333 [0.928; 747.473]	20 [0.916; 436.668]	0.759 [0.478; 1.207]
2017 Jonckheere (23)	0.728 [0.641; 0.804]	0.995 [0.986; 0.999]	487.621 [158.926; 1496.128]	134.241 [46.962; 383.728]	0.275 [0.207; 0.366]
2017 Kumthip-1 (24)	1.000 [0.891; 1.000]	1.000 [0.631; 1.000]	1105 [20.402; 59849.532]	17.727 [1.198; 262.211]	0.016 [0.001; 0.252]
2017 Kumthip-2 (24)	1.000 [0.891; 1.000]	1.000 [0.631; 1.000]	1105 [20.402; 59849.532]	17.727 [1.198; 262.211]	0.016 [0.001; 0.252]
2018 Khamrin (25)	0.895 [0.752; 0.971]	1.000 [0.805; 1.000]	268.333 [13.659; 5271.413]	31.846 [2.066; 490.932]	0.119 [0.05; 0.284]
2018 Sakalkina (26)	0.122 [0.063; 0.208]	1.000 [0.884; 1.000]	8.824 [0.504; 154.392]	7.835 [0.476; 129.096]	0.888 [0.811; 0.972]
2020 Shaha (27)	1.000 [0.692; 1.000]	0.989 [0.940; 1.000]	1253 [47.916; 32765.715]	57.909 [11.782; 284.623]	0.046 [0.003; 0.693]
**Bivariate summary estimates**	**0.609 [0.542; 0.673]**	**0.967 [0.951; 0.978]**	**53.901 [31.316; 92.776]**	**17.082 [11.145; 26.182]**	**0.399 [0.343; 0.464]**

Numbers are pooled estimates with 95% confidence interval; LR+, positive likelihood ratio, LR−, negative likelihood ratio, DOR, diagnostic odds ratio

REFERENCES

1. Okame M, Yan H, Akihara S, Okitsu S, Tani H, Matsuura Y, Ushijima H. 2003. Evaluation of a newly developed immunochromatographic method for detection of norovirus. Kansenshogaku Zasshi 77:637-639. https://doi.org/10.11150/kansenshogakuzasshi1970.77.637.

2. Nguyen TA, Khamrin P, Takanashi S, Le Hoang P, Pham le D, Hoang KT, Satou K, Masuoka Y, Okitsu S, Ushijima H. 2007. Evaluation of immunochromatography tests for detection of rotavirus and norovirus among Vietnamese children with acute gastroenteritis and the emergence of a novel norovirus GII.4 variant. J Trop Pediatr 53:264-269. https://doi.org/10.1093/tropej/fmm021.

3. Khamrin P, Nguyen TA, Phan TG, Satou K, Masuoka Y, Okitsu S, Maneekarn N, Nishio O, Ushijima H. 2008. Evaluation of immunochromatography and commercial enzyme-linked immunosorbent assay for rapid detection of norovirus antigen in stool samples. J Virol Methods 147:360-363. https://doi.org/10.1016/j.jviromet.2007.09.007.

4. Takanashi S, Okame M, Shiota T, Takagi M, Yagyu F, Tung PG, Nishimura S, Katsumata N, Igarashi T, Okitsu S, Ushijima H. 2008. Development of a rapid immunochromatographic test for noroviruses genogroups I and II. J Virol Methods 148:1-8. https://doi.org/10.1016/j.jviromet.2007.10.010.

5. Khamrin P, Takanashi S, Chan-It W, Kobayashi M, Nishimura S, Katsumata N, Okitsu S, Maneekarn N, Nishio O, Ushijima H. 2009. Immunochromatography test for rapid detection of norovirus in fecal specimens. J Virol Methods 157:219-222. https://doi.org/10.1016/j.jviromet.2008.12.012.

6. Mutoh K, Hakamata A, Yagi H, Kurokawa K, Miki N, Kurita I. 2009. Evaluation of new commercial immunochromatography kit for norovirus in feces. Pediatr Int 51:164-166. https://doi.org/10.1111/j.1442-200X.2008.02788.x.

7. Bruins MJ, Wolfhagen MJ, Schirm J, Ruijs GJ. 2010. Evaluation of a rapid immunochromatographic test for the detection of norovirus in stool samples. Eur J Clin Microbiol Infect Dis 29:741-743. https://doi.org/10.1007/s10096-010-0911-5.

8. Kirby A, Gurgel RQ, Dove W, Vieira SC, Cunliffe NA, Cuevas LE. 2010. An evaluation of the RIDASCREEN and IDEIA enzyme immunoassays and the RIDAQUICK immunochromatographic test for the detection of norovirus in faecal specimens. J Clin Virol 49:254-257. https://doi.org/10.1016/j.jcv.2010.08.004.

9. Thongprachum A, Khamrin P, Chaimongkol N, Malasao R, Okitsu S, Mizuguchi M, Maneekarn N, Ushijima H. 2010. Evaluation of an immunochromatography method for rapid detection of noroviruses in clinical specimens in Thailand. J Med Virol 82:2106-2109. https://doi.org/10.1002/jmv.21916.

10. Bruggink LD, Witlox KJ, Sameer R, Catton MG, Marshall JA. 2011. Evaluation of the RIDA(®)QUICK immunochromatographic norovirus detection assay using specimens from Australian gastroenteritis incidents. J Virol Methods 173:121-126. https://doi.org/10.1016/j.jviromet.2011.01.017.

11. Battaglioli G, Nazarian EJ, Lamson D, Musser KA, St George K. 2012. Evaluation of the RIDAQuick norovirus immunochromatographic test kit. J Clin Virol 53:262-264. https://doi.org/10.1016/j.jcv.2011.12.007.

12. Kim HS, Hyun J, Kim JS, Song W, Kang HJ, Lee KM. 2012. Evaluation of the SD Bioline Norovirus rapid immunochromatography test using fecal specimens from Korean gastroenteritis patients. J Virol Methods 186:94-98. https://doi.org/10.1016/j.jviromet.2012.08.014.

13. Park KS, Baek KA, Kim DU, Kwon KS, Bing SH, Park JS, Nam HS, Lee SH, Choi YJ. 2012. Evaluation of a new immunochromatographic assay kit for the rapid detection of norovirus in fecal specimens. Ann Lab Med 32:79-81. https://doi.org/10.3343/alm.2012.32.1.79.

14. Pombubpa K, Kittigul L. 2012. Assessment of a rapid immunochromatographic test for the diagnosis of norovirus gastroenteritis. Eur J Clin Microbiol Infect Dis 31:2379-2383. https://doi.org/10.1007/s10096-012-1579-9.

15. Ambert-Balay K, Pothier P. 2013. Evaluation of 4 immunochromatographic tests for rapid detection of norovirus in faecal samples. Journal of Clinical Virology 56:278-282. https://doi.org/https://doi.org/10.1016/j.jcv.2012.11.001.

16. Bruggink LD, Catton MG, Marshall JA. 2013. Evaluation of the Bioline Standard Diagnostics SD immunochromatographic norovirus detection kit using fecal specimens from Australian gastroenteritis incidents. Diagnostic Microbiology and Infectious Disease 76:147-152. https://doi.org/https://doi.org/10.1016/j.diagmicrobio.2013.02.018.

17. Kas MP, Maure T, Soli KW, Umezaki M, Morita A, Bebes S, Jonduo MH, Larkins JA, Luang-Suarkia D, Siba PM, Greenhill AR, Horwood PF. 2013. Evaluation of a rapid immunochromatographic assay for the detection of rotavirus, norovirus and adenovirus from children hospitalized with acute watery diarrhea. P N G Med J 56:141-144.

18. Bruggink LD, Dunbar NL, Marshall JA. 2015. Evaluation of the updated RIDA® QUICK (Version N1402) immunochromatographic assay for the detection of norovirus in clinical specimens. J Virol Methods 223:82-87. https://doi.org/10.1016/j.jviromet.2015.07.019.

19. Vyas K, Atkinson C, Clark DA, Irish D. 2015. Comparison of five commercially available immunochromatographic tests for the detection of norovirus in faecal specimens. J Hosp Infect 91:176-178. https://doi.org/10.1016/j.jhin.2015.06.013.

20. Hosoda T, Uehara Y, Matsuda N, Kawase Y, Tanei M, Haba Y, Nakamura A, Tabe Y, Naito T, Ohsaka A. 2016. Performance Evaluation of a Novel Fully Automated Real-Time Reverse Transcriptase-Polymerase Chain Reaction Kit for the Detection of Norovirus. Rinsho Byori 64:1347-1352.

21. Sharaf HE, Morsi SS, Gerges MA. 2016. Performance characteristics of enzyme linked immunosorbent assay and rapid immunochromatographic test for routine screening of human norovirus. African Journal of Clinical and Experimental Microbiology 17:205-212. https://doi.org/10.4314/ajcem.v17i3.8.

22. Gaspard P, Pothier P, Roth C, Larocca S, Heck B, Ambert-Balay K. 2017. Viral prevalence and laboratory investigations of gastroenteritis in institutions for dependent people. Med Mal Infect 47:546-553. https://doi.org/10.1016/j.medmal.2017.09.007.

23. Jonckheere S, Botteldoorn N, Vandecandelaere P, Frans J, Laffut W, Coppens G, Vankeerberghen A, De Beenhouwer H. 2017. Multicenter evaluation of the revised RIDA® QUICK test (N1402) for rapid detection of norovirus in a diagnostic laboratory setting. Diagnostic Microbiology and Infectious Disease 88:31-35. https://doi.org/https://doi.org/10.1016/j.diagmicrobio.2017.02.006.

24. Kumthip K, Khamrin P, Saikruang W, Supadej K, Ushijima H, Maneekarn N. 2017. Comparative Evaluation of Norovirus Infection in Children with Acute Gastroenteritis by Rapid Immunochromatographic Test, RT-PCR and Real-time RT-PCR. J Trop Pediatr 63:468-475. https://doi.org/10.1093/tropej/fmx014.

25. Khamrin P, Kumthip K, Thongprachum A, Takanashi S, Okitsu S, Maneekarn N, Hayakawa S, Ushijima H. 2018. Evaluation of Immunochromatographic Test for Dual Detection of Noroviruses and Group A Rotaviruses in Stool Samples. Clin Lab 64:793-796. https://doi.org/10.7754/Clin.Lab.2017.171201.

26. Sakalkina EV, Parkina NV, Olneva TA, Tagirova ZG, Podkolzin AT, Shipulin GA. 2018. A Comparison of Diagnostics Kits Used in Russia for Identification of Antigens of Noroviruses. Molecular Genetics, Microbiology and Virology 33:44-48. https://doi.org/10.3103/S0891416818010111.

27. Shaha M, Sifat SF, Al Mamun M, Billah MB, Sharif N, Nobel NU, Parvez AK, Talukder AA, Nomura A, Ushijima H, Dey SK. 2020. Comparative evaluation of sensitivity and specificity of immunochromatography kit for the rapid detection of norovirus and rotavirus in Bangladesh [version 2]. F1000Research 8. https://doi.org/10.12688/F1000RESEARCH.17362.1.
