# Supplementary material for: Diagnostic Accuracy of Immunochromatographic Tests for the Detection of Norovirus in Stool Specimens: a Systematic Review and Meta-Analysis
Source: Microbiol Spectr. 2021 Jul 7;9(1):10.1128/spectrum.00467-21. doi: 10.1128/spectrum.00467-21 (PMC8552764; doi:10.1128/spectrum.00467-21)
Supplement: Supplemental file 2 — Supplemental material. Download SPECTRUM00467-21_Supp_2_seq8.pdf, PDF file, 0.4 MB [file spectrum00467-21_supp_2_seq8.pdf]

TABLE S2. Meta-regression for diagnostic odds ratio

	Estimate (SE)	p-value
**Population**		**<.001**
Adults vs. Children	-2.055 (0.880)	0.020
Children and adults vs. Children	-1.246 (0.553)	0.037
NA vs. Children	-1.246 (0.553)	0.024
**Included NV genogroup**		**0.653**
GI vs. GII	-1.283 (2.255)	0.569
GI and GII vs. GII	-0.756 (0.684)	0.270
NA vs. GII	-1.540 (1.850)	0.405
**Type of specimen**		**0.043**
Unfrozen vs. Frozen	-0.794 (1.029)	0.440
NA vs. Frozen	-1.588 (0.637)	0.013
**Brand**		**0.363**
etc. vs. RIDAQUICK	-0.530 (0.582)	0.363
**Reference test**		**0.250**
Real-time RT-PCR vs. RT-PCR	-0.707 (0.615)	0.250

NA, not available; SE, standard error.

*Children and adults were defined as younger and older than 18 years old, respectively.
