# Supplementary material for: Diagnostic Accuracy of Immunochromatographic Tests for the Detection of Norovirus in Stool Specimens: a Systematic Review and Meta-Analysis
Source: Microbiol Spectr. 2021 Jul 7;9(1):10.1128/spectrum.00467-21. doi: 10.1128/spectrum.00467-21 (PMC8552764; doi:10.1128/spectrum.00467-21)

FIGURE S2. Forest plot of the specificity estimates of immunochromatographic tests for diagnosing norovirus infection. Numbers are pooled estimates with 95% confidence interval. Horizontal lines indicate 95% CIs.

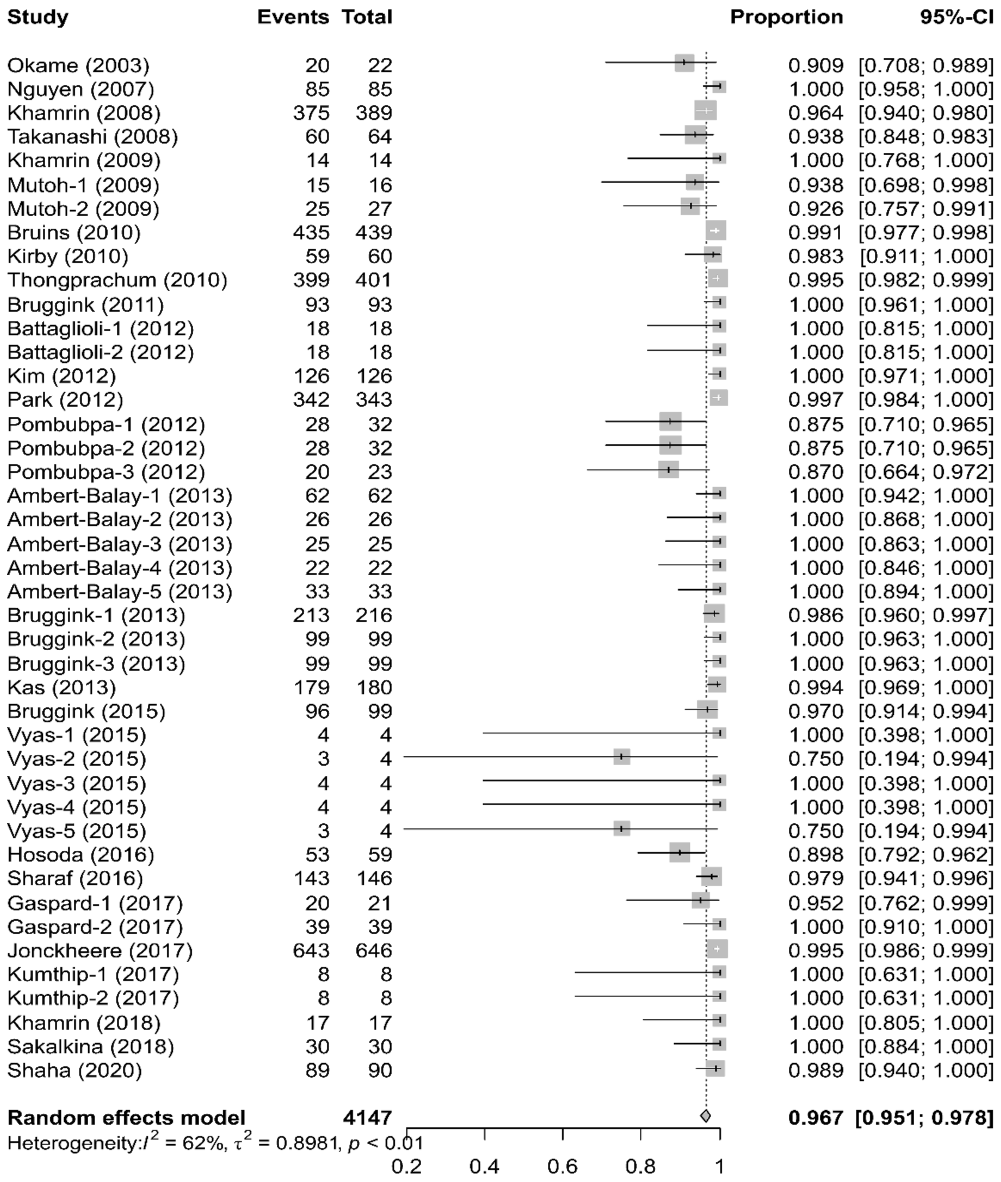

Supplement: Supplemental file 4 — Supplemental material. Download SPECTRUM00467-21_Supp_4_seq10.pdf, PDF file, 0.5 MB [file spectrum00467-21_supp_4_seq10.pdf]
