# Supplementary figures and images for: Diagnostic Accuracy of Immunochromatographic Tests for the Detection of Norovirus in Stool Specimens: a Systematic Review and Meta-Analysis
Source: Microbiol Spectr. 2021 Jul 7;9(1):10.1128/spectrum.00467-21. doi: 10.1128/spectrum.00467-21 (PMC8552764; doi:10.1128/spectrum.00467-21)

FIGURE S3. Contour-enhanced funnel plot for publication bias detection

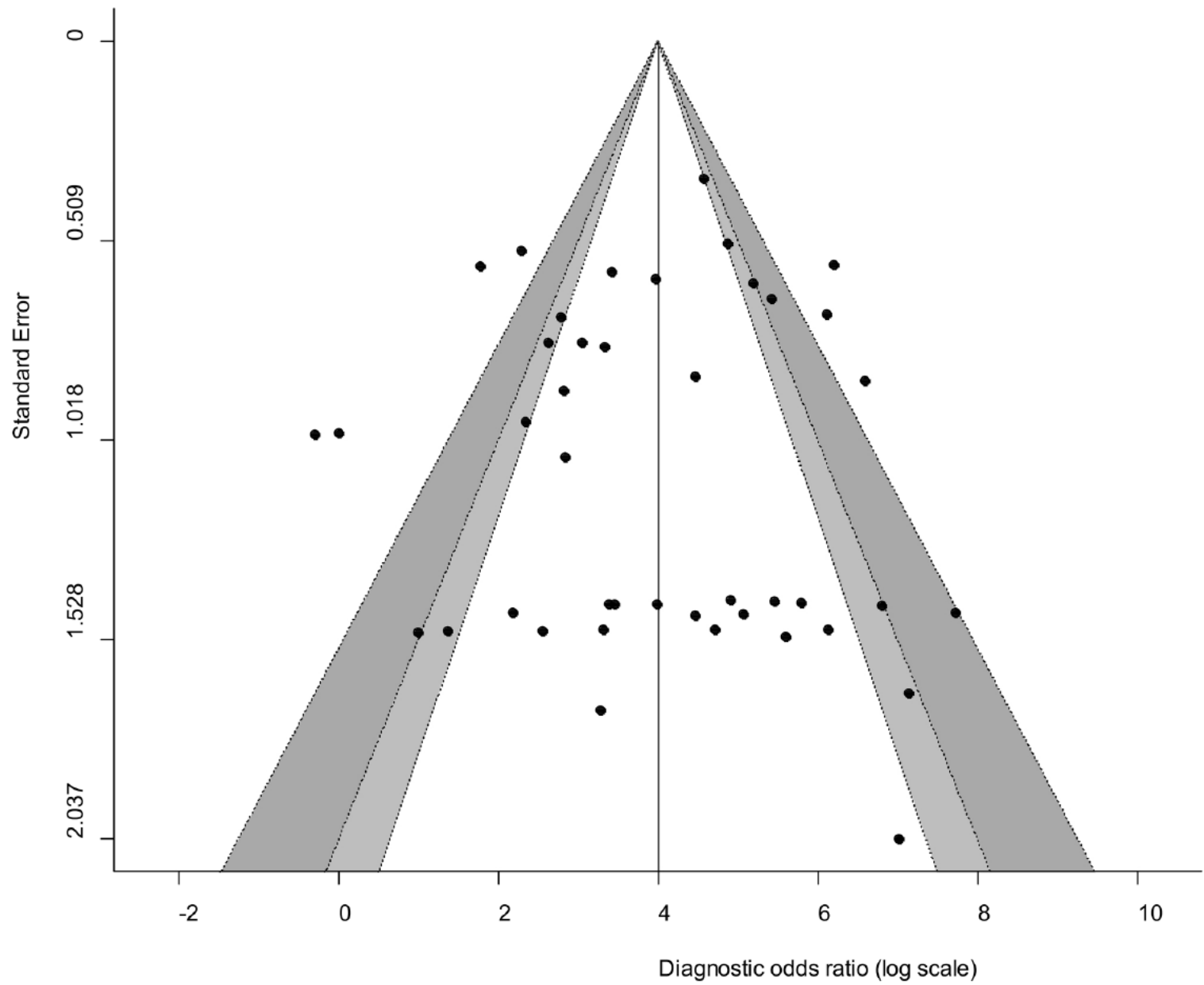

Supplement: Supplemental file 5 — Supplemental material. Download SPECTRUM00467-21_Supp_S3_seq11.pdf, PDF file, 0.4 MB [file spectrum00467-21_supp_s3_seq11.pdf]
